# The efficacy of antipsychotics in the treatment of physical aggressive behavior in patients with dementia in nursing homes

**DOI:** 10.1177/20451253221097452

**Published:** 2022-05-16

**Authors:** Sina Nawzad, Wiepke Cahn, Heshu Abdullah-Koolmees

**Affiliations:** Department of Clinical Pharmacy, University Medical Center Utrecht, Utrecht, The Netherlands; Department of Psychiatry, University Medical Center Utrecht, Utrecht, The Netherlands; Department of Pharmacoepidemiology and Clinical Pharmacology, Utrecht Institute for Pharmaceutical Sciences, Faculty of Science, Utrecht University, Postbus 85500, 3508 GA Utrecht, The Netherlands; Department of Clinical Pharmacy, University Medical Center Utrecht, Utrecht, The Netherlands; Department of Clinical Pharmacology and Pharmacy, VU University Medical Center, Amsterdam, The Netherlands

**Keywords:** aggressive behavior, *Cohen-Mansfield Agitation Inventory* (CMAI) scale, antipsychotics, dementia, neuropsychiatric symptoms

## Abstract

Patients with dementia often suffer from behavioral changes. A common behavioral change is acute physical aggressive behavior which is the most distressing change. This can lead to harm, which is especially problematic in nursing homes. Despite the serious safety concerns, antipsychotics are often prescribed to combat this problem. This article is aimed to review the evidence of the efficacy of utilizing antipsychotics in acutely treating physical aggressive behavior in patients with dementia in nursing homes. Therefore, a systematic literature search was performed. The results demonstrated that a meta-analysis confirmed statistically significant reduction in physical aggression when risperidone was compared to placebo. However, a randomized controlled trial showed no change in physical aggressive behavior between quetiapine and placebo. More research is needed to fully investigate the benefits of physical aggressive behavior and safety concerns of all the antipsychotics in patients with dementia in nursing homes.

## Introduction

Patients with dementia often suffer from behavioral changes, referred to as neuropsychiatric symptoms (NPSs) or behavioral and psychological symptoms of dementia (BPSD).^1^ Up to 90% of patients with dementia develop BPSD.^2^ BPSD include uninhibited behavior, agitation, and aggression. BPSD are very distressing for patients. Acute physical aggression can even lead to self-harm and harm to others around them.^3,4^ This is especially problematic in nursing homes and needs to be managed both diligently and expeditiously. Non-pharmacological interventions, which is first-line treatment, is not effective in managing these symptoms for every patient, their benefits are typically limited to milder severity of aggression. Many patients fail these interventions after initial success.^4^ Therefore, a wide variety of pharmacotherapies are often prescribed off-label to combat this problem. There are currently no *‘USA Food and Drug Administration’* (FDA)-approved medications indicated for the treatment of aggression in patients with dementia. The European Union, however, has only approved the antipsychotic risperidone for the treatment of persistent aggression in patients with moderate to severe Alzheimer’s dementia.^5^ Thus, there are no medications on the market indicated to treat acute aggression in this patient population. Nevertheless, antipsychotics are often used for this indication. The FDA issued warnings in 2003 and 2005 regarding the use of antipsychotics in patients with dementia.^2^ Post-marketing surveillance demonstrated serious adverse events associated with the use of antipsychotics, including increased mortality. Evidence form clinical trials demonstrated a 1.5- to 1.8-fold increased mortality.^6,7^ Despite these safety concerns, antipsychotics are still being widely prescribed off-label. Recently, Zuidema *et al.*^4^ published a practice guideline regarding the prescribing of antipsychotics to patients with dementia in nursing homes. This guideline is intended to further aid clinicians in the treatment process. However, it is not evidence-based. This article aims to review the evidence of the efficacy of utilizing antipsychotics in the acute treatment of non-psychotic-induced dementia-related physical aggressive behavior in patients with dementia in nursing homes.

## Objective

To investigate the efficacy of antipsychotics in acutely treating physical aggressive behavior in patients with dementia in nursing homes.

## Methods

A systematic literature search was performed in the following databases: PubMed, Cochrane, and Embase. The initial search was performed on 29 October 2018, and the search was updated on 24 December 2021. The targeted search was built with a ‘patient intervention comparison outcome’ (PICO)-model based on the following question: what is the efficacy of antipsychotics for the treatment of acute non-psychotic-induced aggressive behavior in patients with dementia in nursing homes? The population included patients in nursing homes with different forms of dementia and non-psychotic-induced acute aggressive behavior. The intervention was comparing antipsychotic to placebo. The outcome was efficacy, such as a change in manifestation of aggressive behavior or a change in the frequency of aggressive behavior. Therefore, the search included the synonyms of dementia, aggressive behavior, and antipsychotics found in MESH/Emtree terms, supplementary concept title, and abstract. The label languages (English), species (humans), and text availability (full text) were also added to the search criteria (see Supplemental Appendix 1 for exact search terms). All search results were first manually checked based on the relevance by reading the title and the abstract. Article types of observational studies or randomized clinical trials (RCTs) that compared antipsychotics with placebo or meta-analysis investigating the efficacy of antipsychotic drugs were included. Article types other than the aforementioned types were excluded considering RCTs and meta-analysis of RCTs as the ‘best evidence’ in the study hierarchy of therapeutic trials.^8^ In addition, articles were excluded if patients with other disease states were included (e.g. Schizophrenia, Parkinson’s disease, psychosis, and bipolar disease), utilized interventions other than antipsychotics (e.g. antidepressants), and contained outcomes other than efficacy (e.g. results with only safety data). These remaining articles were screened by the first author by inclusion criteria and outcome results. Articles that included patients without the diagnosis of dementia according to the *Diagnostic and Statistical Manual of Mental Disorders IV* (*DSM*-IV) in nursing homes or articles that did not specifically address acute aggressive behavior and had no separate data on aggressive behavior were also excluded. Quality assessment of the included articles will be performed using the Consolidated Standards of Reporting Trials (CONSORT) checklist^9^ for RCTs, the Preferred Reporting Items for Systematic Reviews and Meta-Analysis (PRISMA) checklist^10^ for meta-analysis, and the observational study quality evaluation (OSQE) checklist^11^ for observational studies.

## Results

Overall, the initial search yielded 284 articles, and the updated search resulted in 294 articles (Supplemental Appendix 2). For both searches, only 104 articles remained after excluding article types other than observational studies or RCTs that compared antipsychotics with placebo or meta-analysis. After reading the titles and abstracts, 35 articles remained based on the patient’s disease states, utilized interventions, and outcomes. These articles were further full text screened based on their inclusion criteria (i.e. patients with dementia in nursing homes) and reported outcomes (i.e. data on physical aggressive behavior), narrowing the search results down to five articles (Supplemental Appendix 2). In total, only five articles evaluated acute non-psychotic-induced aggressive behavior in patients with dementia that were residing in nursing homes and being treated with antipsychotics. One of these articles was a meta-analysis^12^ that contained three RCTs,^13–15^ which were already a part of the final search results and were therefore excluded.

Ultimately, there was one RCT and one meta-analysis analyzed in this study,^12,16^ see Table 1 and Tables S1 and S2 of the Supplemental Appendix 3 for relevant trial design and results. Quality assessment of these articles was performed and can be found in both Tables S3 and S4 of Supplemental Appendix 4. The outcome, physical aggressive behavior, was assessed using the *Cohen-Mansfield Agitation Inventory* (CMAI) scale in both articles. CMAI is a frequency rating scale containing 29-items to assess various aggressive and agitated behaviors reported as the CMAI total score. The CMAI score can also be divided into subscale scores, such as verbal or physical-aggression subscale score. The outcome in the studies was reported as the observed mean change in CMAI physical-aggression subscale score from baseline to endpoint.^12,17^ The pooled data of the meta-analysis of De Deyn *et al.*^12^ demonstrated a statistically significant reduction in physical aggression when comparing risperidone to placebo. However, Zhong *et al.*^16^ demonstrated no difference in mean change (from baseline to evaluated points) in CMAI physical-aggression subscale score between quetiapine and placebo (Table 2).

**Table 1.** Summary of trial design.^12–16^

Study	Objective	Patient selection	Trial design	Efficacy assessment
Meta-analysis De Deyn *et al.*^12^ of the trails: Brodaty *et al.*,^13^ De Deyn *et al.*^14^ and Katz *et al.*^15^	Pooled data analysis that examined the efficacy of risperidone for the treatment of aggression associated with dementia in elderly nursing home residents.	Patients (aged > 55 years) lived in an institution and had to have been diagnosed according to *DSM*-IV criteria with dementia of the Alzheimer’s type, vascular dementia, or a combination of the two (i.e. mixed dementia), together with BPSD that were at least mildly troubling to their caregivers or dangerous to themselves.	12-week, phase IIIA, double-blind, placebo-controlled, parallel-group preceded by 1-week single-blind washout periods during which all other psychotropic medications were discontinued.	Change in CMAI physical-aggression score from baseline to evaluated points (weeks 4, 8, 12, and at endpoint).
Zhong *et al.*^16^	To evaluate the efficacy of two fixed doses of quetiapine compared with placebo in treating agitation in patients with dementia.	Patients (aged > 55 years) were residents of nursing homes and assisted living facilities. They had diagnoses of probable or possible AD or vascular dementia according to *DSM*-IV or NINCDS/ADRDA and documented clinical symptoms of agitation.	10-week, randomized, multicenter, double-blind, placebo-controlled, fixed-dose (quetiapine 200 and 100 mg/day) study.	Change in CMAI physical-aggression score from baseline to evaluated points (weeks 1, 2, 4, 6, and 10 or at study withdrawal)

AD: Alzheimer’s disease; BPSD: behavioral and psychological symptoms of dementia; CMAI: Cohen-Mansfield agitation inventory; *DSM*-IV: Diagnostic and Statistical Manual of Mental Disorders–Fourth Edition; NINCDS/ADRDA: National Institute of Neurological and Communicative Disorders and Stroke/the Alzheimer’s Disease and Related Disorders Association.

**Table 2.** Results of the CMAI physical-aggression subscale score: mean change from baseline to endpoint.^12,16^.

		Intervention			Placebo			Overall *p* value
Study	Intervention, mean dose (mg/day)	*N*	*M* (SE)	95% CI	*N*	*M*	95% CI	
De Deyn *et al.*^12^	Risperidone, 1.0	713	−3.5 (0.34)	−4.21; −2.87	426	−1.4 (0.48)	−2.32; −3.2	<0.001
Zhong *et al.*^16^	Quetiapine, 100	120	−3.2 (0.9)	−1.7; 2.2	94	−2.9 (0.8)	Missing data	0.796
	Quetiapine, 200	114	−3.7 (0.9)	−2.0; 1.9				0.976

CI: confidence Interval; CMAI: Cohen–Mansfield agitation inventory; SE, standard error.

## Discussion

This systematic literature study suggests that there is some evidence for the efficacy of risperidone in treating acute physical aggressive behavior in patients with dementia residing in nursing homes. Quetiapine, however, was not associated with statistically significant benefits; thus, the results of the included studies show no evidence for the efficacy of quetiapine in treating acute physical aggressive behavior in patients with dementia in nursing homes. Other antipsychotics are not investigated to specifically treat non-psychosis-induced acute aggressive behavior in this specific patient population, namely patients with dementia residing in nursing homes. A double-blind study conducted by Meehan *et al.*^18^ investigated the efficacy of rapid-acting intramuscular olanzapine in treating agitation associated with Alzheimer’s disease and/or vascular dementia (Supplemental Appendix 3). They concluded that olanzapine showed superiority to placebo on the CMAI score. A rapid-acting intervention is precisely important in treating patient with acute physical aggressive behavior in nursing homes. However, this study did not report results on the psychical aggressive behavior subscale score separately. Thus, olanzapine may be promising to treat psychical aggressive behavior specifically in these patients who need acute treatment, although not all patients of this trail were nursing home residents. In addition, further research is needed to fully determine the efficacy of all the antipsychotic agents.

The strength of our study was the high level of evidence: results were obtained through a meta-analysis study and a RCT. The meta-analysis conducted by De Deyn *et al.*^12^ contained three RCTs, which were similar in design, patient characteristics, efficacy scale, intervention, and treatment duration. Therefore, pooling the data was justified and useful since a larger patient population allows more precise estimates of the effect size.

One limitation of the De Deyn *et al.*^12^ study was their patient population. Their study included patients with psychosis (approximately 60%), which was part of our exclusion criteria. However, they performed a subgroup analysis that showed significant improvement in aggressive behavior in patients who did not have psychosis. This ultimately indicates that risperidone may improve symptoms of aggression independent of its antipsychotic effect.

Currently, there is some evidence supporting the use of risperidone for treating acute physical aggressive behavior in patients with dementia residing in nursing homes. However, more research is needed on efficacy and safety. Thus, the recommendation for daily practice is to keep the use of antipsychotics in the treatment of aggressive behavior in patients with dementia in nursing homes to a minimum. When strictly necessary, risperidone should be utilized in this setting, despite its safety concerns, until we gain more insight into the safety and efficacy of utilizing these antipsychotics.

## Conclusion

The objective of this study was to evaluate the efficacy of antipsychotic agents compared to placebo in treating acute physical aggressive behavior in patients with dementia residing in nursing homes. Results of the meta-analysis of De Deyn *et al.*^12^ confirmed that risperidone might be an effective treatment for reducing physical aggressive behavior in this patient population. However, the randomized controlled trial of Zhong *et al.*^16^ showed no change in physical aggressive behavior between quetiapine and placebo. Currently, only risperidone is proven to be effective in one meta-analysis for this indication and patient population. In conclusion, more research is needed to fully investigate the benefits of all the antipsychotics in treating acute physical aggressive behavior in patients with dementia residing in nursing homes.

## Supplemental Material

sj-docx-1-tpp-10.1177_20451253221097452 – Supplemental material for The efficacy of antipsychotics in the treatment of physical aggressive behavior in patients with dementia in nursing homes Click here for additional data file. Supplemental material, sj-docx-1-tpp-10.1177_20451253221097452 for The efficacy of antipsychotics in the treatment of physical aggressive behavior in patients with dementia in nursing homes by Sina Nawzad, Wiepke Cahn and Heshu Abdullah-Koolmees in Therapeutic Advances in Psychopharmacology
