# Supplementary material for: The efficacy of antipsychotics in the treatment of physical aggressive behavior in patients with dementia in nursing homes
Source: Ther Adv Psychopharmacol. 2022 May 16;12:20451253221097452. doi: 10.1177/20451253221097452 (PMC9118396; doi:10.1177/20451253221097452)
Supplement: sj-docx-1-tpp-10.1177_20451253221097452 – Supplemental material for The efficacy of antipsychotics in the treatment of physical aggressive behavior in patients with dementia in nursing homes [file sj-docx-1-tpp-10.1177_20451253221097452.docx]

**The efficacy of antipsychotics in the treatment of physical aggressive behavior in patients with dementia in nursing homes.**

Nawzad S. MD, Pharm D

Wiepke Cahn, PhD, MD

Abdullah-Koolmees H. PhD, Pharm D

**Appendix 1 search terms**

- Search PubMed:

(((((((((((((((((((((((((Risperidone[Title/Abstract]) OR Risperidone[Supplementary Concept]) OR Quetiapine[Title/Abstract]) OR Quetiapine[Supplementary Concept]) OR Paliperidone[Supplementary Concept]) OR Paliperidone[Title/Abstract]) OR Olanzapine[Supplementary Concept]) OR Olanzapine[Title/Abstract]) OR Clozapine[Title/Abstract]) OR Clozapine[Supplementary Concept]) OR Aripiprazole[Title/Abstract]) OR Aripiprazole[Supplementary Concept]) OR Haloperidol[Supplementary Concept]) OR Haloperidol[Title/Abstract]) OR Fluphenazine[Supplementary Concept]) OR Fluphenazine[Title/Abstract]) OR droperidol[Title/Abstract]) OR droperidol[Title/Abstract]) OR Chlorpromazine[Supplementary Concept]) OR Chlorpromazine[Title/Abstract]) OR "Antipsychotic Agents"[Mesh]) OR neuroleptics) OR Antipsychoti*[Title/Abstract])) AND ((((((((((((uninhibit*[Title/Abstract]) AND behavior[Title/Abstract])) OR ((uninhibited[Title/Abstract]) AND behavior[Title/Abstract])) OR ((neuropsychia*[Title/Abstract]) AND symptom*[Title/Abstract])) OR neuropsychia*[Title/Abstract]) OR agitation[Title/Abstract]) OR ((neuropsychiatric[Title/Abstract]) AND symptoms[Title/Abstract])) OR aggression[Title/Abstract]) OR neuropsychiatric symptoms[Title/Abstract]) OR problem behavior[Title/Abstract]) OR "Aggression"[Mesh])) AND (((((Lewy Body Disease[Title/Abstract]) OR Frontotemporal Lobar Degeneration[Title/Abstract]) OR dement*[Title/Abstract]) OR dementia[Title/Abstract]) OR "Dementia"[Mesh]) AND ("loattrfull text"[sb] AND "humans"[MeSH Terms] AND English[lang]) AND (efficacy[Title/Abstract]). The search performed on December 24^th^, 2021 resulted in 238 records.

- Search Cochrane:

The Cochrane database was browsed by Cochrane Review Group ‘Dementia and Cognitive Improvement’, topic: mental health, dementia & cognition, interventions aimed at manifestations of the disease. The search performed on December 24^th^, 2021 resulted in 32 records.

- Search Embase:

(('dementia'/exp OR 'alzheimer disease'/exp OR 'alzeimer disease' OR 'alzeimer`s disease' OR 'alzeimers disease' OR 'alzheimer dementia' OR 'alzheimer disease' OR 'alzheimers disease' OR 'alzheimer fibrillary change' OR 'alzheimer fibrillary lesion' OR 'alzheimer neurofibrillary change' OR 'alzheimer neurofibrillary degeneration' OR 'alzheimer neuron degeneration' OR 'alzheimer perusini disease' OR 'alzheimer sclerosis' OR 'alzheimer syndrome' OR 'alzheimer`s disease' OR 'cortical sclerosis, diffuse' OR 'dementia, alzheimer' OR 'diffuse cortical sclerosis' OR 'late onset alzheimer disease' OR 'frontotemporal dementia'/exp OR 'ftd (frontotemporal dementia)' OR 'ftld' OR 'pick complex' OR 'pick`s complex' OR 'dementia, frontotemporal' OR 'frontal dementia' OR 'frontal lobe dementia' OR 'frontotemporal dementia' OR 'frontotemporal dementias' OR 'frontotemporal lobar degeneration' OR 'diffuse lewy body disease'/exp OR 'dlb (dementia with lewy bodies)' OR 'dlbd' OR 'lbd (lewy body disease)' OR 'lewy body dementia' OR 'lewy body dementias' OR 'lewy body disease' OR 'lewy body diseases' OR 'dementia with lewy bodies' OR 'dementia with lewy body' OR 'diffuse lewy body disease') AND ('problem behavior'/exp OR 'behavior problem' OR 'behavioral problem' OR 'behavioral problems' OR 'behaviour problem' OR 'behavioural problem' OR 'behavioural problems' OR 'behavoural problems' OR 'problem behavior' OR 'problem behaviors' OR 'problem behaviour' OR 'problem behaviours' OR 'problematic behavior' OR 'problematic behaviors' OR 'problematic behaviour' OR 'problematic behaviours' OR 'problembehavior') OR 'aggression'/exp OR 'aggression' OR 'aggressive attitude' OR 'aggressive behavior' OR 'aggressive behaviour' OR 'aggressive reaction' OR 'behavior, aggressive' OR 'behaviour, aggressive' OR 'counter aggression' OR 'counteraggression' OR 'agitation'/exp OR 'agitation') AND ('neuroleptic agent'/exp OR 'antipsychotic agent' OR 'antipsychotic agents' OR 'antipsychotic agents, butyrophenone' OR 'antipsychotic agents, phenothiazine' OR 'antipsychotic drug' OR 'antipsychotics' OR 'butyrophenone tranquilizers' OR 'classical antipsychotic' OR 'classical antipsychotic agent' OR 'classical antipsychotic drug' OR 'long acting neuroleptic' OR 'major tranquilizer' OR 'major tranquillizer' OR 'neuroleptic' OR 'neuroleptic agent' OR 'neuroleptic drug' OR 'neurolepticum' OR 'phenothiazine tranquilizers' OR 'tranquilizer, major' OR 'tranquilizing agents, major' OR 'typical antipsychotic' OR 'typical antipsychotic agent' OR 'typical antipsychotic drug' OR 'typical neuroleptic' OR 'typical neuroleptic agent' OR 'typical neuroleptic drug') AND ('placebo'/exp OR 'placebo' OR 'placebo gel' OR 'placebos') AND ('therapy'/exp OR 'combination therapy' OR 'disease therapy' OR 'disease treatment' OR 'diseases treatment' OR 'disorder treatment' OR 'disorders treatment' OR 'efficacy, therapeutic' OR 'illness treatment' OR 'medical therapy' OR 'medical treatment' OR 'multiple therapy' OR 'polytherapy' OR 'somatotherapy' OR 'therapeutic action' OR 'therapeutic efficacy' OR 'therapeutic trial' OR 'therapeutic trials' OR 'therapeutics' OR 'therapy' OR 'therapy, medical' OR 'treatment effectiveness' OR 'treatment efficacy' OR 'treatment, medical') AND 'safety'/exp. The search performed on December 24^th^, 2021 resulted in 24 records.

**Appendix 2 Reference list of search results**

1. De Deyn PP, Katz IR, Brodaty H, Lyons B, Greenspan A, Burns A. Management of agitation, aggression, and psychosis associated with dementia: a pooled analysis including three randomized, placebo-controlled double-blind trials in nursing home residents treated with risperidone. Clin Neurol Neurosurg. 2005 Oct;107(6):497-508.
2. Brodaty H, Ames D, Snowdon J, Woodward M, Kirwan J, Clarnette R, Lee E, Lyons B, Grossman F. A randomized placebo-controlled trial of risperidone for the treatment of aggression, agitation, and psychosis of dementia. J Clin Psychiatry. 2003 Feb;64(2):134-43.
3. De Deyn PP, Rabheru K, Rasmussen A, Bocksberger JP, Dautzenberg PL, Eriksson S, Lawlor BA. A randomized trial of risperidone, placebo, and haloperidol for behavioral symptoms of dementia. Neurology. 1999 Sep 22;53(5):946-55.
4. Katz IR, Jeste DV, Mintzer JE, Clyde C, Napolitano J, Brecher M. Comparison of risperidone and placebo for psychosis and behavioral disturbances associated with dementia: a randomized, double-blind trial. Risperidone Study Group. J Clin Psychiatry. 1999 Feb;60(2):107-15.
5. Zhong KX, Tariot PN, Mintzer J, Minkwitz MC, Devine NA. Quetiapine to treat agitation in dementia: a randomized, double-blind, placebo-controlled study. Curr Alzheimer Res. 2007 Feb;4(1):81-93.

**Appendix 3 Summery of the Trial Design and results of Meehan *et al.***

Table S1 Summary of Trial Design of the study conducted by Meehan *et al.*^15^

| **Objective** | **Patient selection** | **Trial design** | **Efficacy assessment** |
| --- | --- | --- | --- |
| To investigate the efficacy of intramuscular injection of olanzapine in the treatment of agitation associated with dementia. | Patients (age > 55y) were hospitalized or nursing home residents. They had diagnoses of probable or possible AD, vascular dementia or both (mixed dementia) according to DSM-IV or NINCDS/ADRDA and documented clinical symptoms of agitation. | A randomized, multicenter, double-blind, placebo-controlled parallel study. Study period began with initiation of preliminary evaluations for the first injection and continued for 24 h. | Change in CMAI physical-aggression score from the first dose to 2 h, and 24 h post first injection. |

Abbreviations: AD: Alzheimer’s disease, BPSD: behavioral and psychological symptoms of dementia CMAI: Cohen-Mansfield agitation inventory. DSM-IV: Diagnostic and Statistical Manual of Mental Disorders IV, NINCDS/ADRDA: National Institute of Neurological and Communicative Disorders and Stroke/the Alzheimer's Disease and Related Disorders Association.

Table S2 Results of the total Cohen-Mansfield agitation inventory (CMAI) score

|  | Intervention | | Placebo | | Overall p-value |
| --- | --- | --- | --- | --- | --- |
| Intervention, mean dose | N | Mean (SD) | N | Mean (SD) |  |
| Olanzapine, 2.5 mg IM | 71 | −3.8 (2.9)* and −2.8 (3.2)** | 67 | −2.8 (3.4)* and −2.2 (3.6)** | >0.05 |
| Olanzapine, 5 mg IM | 66 | −4.0 (3.9)* and −3.4 (3.9)** |  |  | <0.05* and >0.05** |

Abbreviations: IM: Intramuscular Injection, SD: standard deviation. * Mean Change from Baseline at 2 Hours Post First Intramuscular Injection. ** Mean Change from Baseline at 24 Hours Post First Intramuscular Injection.

**Appendix 4 Quality assessment of the included articles**

Table S3 Results of the quality assessment of Zhong *et al.^13^* by using the Consolidated Standards of Reporting Trials (CONSORT) checklist^16^

| Section/Topic | Item No | Checklist item | Reported on page No |
| --- | --- | --- | --- |
| Title and abstract | | | |
|  | 1a | Identification as a randomised trial in the title | + p. 81 |
|  | 1b | Structured summary of trial design, methods, results, and conclusions | + p. 81 |
| Introduction | | | |
| Background and objectives | 2a | Scientific background and explanation of rationale | + p.81 |
|  | 2b | Specific objectives or hypotheses | + p. 82 |
| Methods | | | |
| Trial design | 3a | Description of trial design (such as parallel, factorial) including allocation ratio | + p. 82 |
|  | 3b | Important changes to methods after trial commencement (such as eligibility criteria), with reasons | + p. 82 |
| Participants | 4a | Eligibility criteria for participants | + p. 82 |
|  | 4b | Settings and locations where the data were collected | + p. 82 |
| Interventions | 5 | The interventions for each group with sufficient details to allow replication, including how and when they were actually administered | + p. 82 |
| Outcomes | 6a | Completely defined pre-specified primary and secondary outcome measures, including how and when they were assessed | + p. 82 |
|  | 6b | Any changes to trial outcomes after the trial commenced, with reasons | + p. 82 |
| Sample size | 7a | How sample size was determined | + p. 83 |
|  | 7b | When applicable, explanation of any interim analyses and stopping guidelines | + p. 83 |
| Randomisation: |  |  |  |
| Sequence generation | 8a | Method used to generate the random allocation sequence | + p. 82 |
|  | 8b | Type of randomisation; details of any restriction (such as blocking and block size) | + p. 82, random block size of 8, using random seed and treatment allocation ratios of 3:3:2 |
| Allocation concealment mechanism | 9 | Mechanism used to implement the random allocation sequence (such as sequentially numbered containers), describing any steps taken to conceal the sequence until interventions were assigned | + p. 82 |
| Implementation | 10 | Who generated the random allocation sequence, who enrolled participants, and who assigned participants to interventions | + p. 82, centralized randomization schedule, blinding maintained by sponsors randomization group |
| Blinding | 11a | If done, who was blinded after assignment to interventions (for example, participants, care providers, those assessing outcomes) and how | +/- p. 82 Study states to be double-blind, but no further information given on how personnel is blinded. |
|  | 11b | If relevant, description of the similarity of interventions | + p. 82 Medication in blister wallets, same number, color, size and shape of tablets. |
| Statistical methods | 12a | Statistical methods used to compare groups for primary and secondary outcomes | + p. 83 |
|  | 12b | Methods for additional analyses, such as subgroup analyses and adjusted analyses | + p. 83 |
| Results | | | |
| Participant flow (a diagram is strongly recommended) | 13a | For each group, the numbers of participants who were randomly assigned, received intended treatment, and were analysed for the primary outcome | + p. 83 |
|  | 13b | For each group, losses and exclusions after randomisation, together with reasons | + p. 83 |
| Recruitment | 14a | Dates defining the periods of recruitment and follow-up | + p. 83 |
|  | 14b | Why the trial ended or was stopped | N/A |
| Baseline data | 15 | A table showing baseline demographic and clinical characteristics for each group | + p. 84 |
| Numbers analysed | 16 | For each group, number of participants (denominator) included in each analysis and whether the analysis was by original assigned groups | + p. 84 |
| Outcomes and estimation | 17a | For each primary and secondary outcome, results for each group, and the estimated effect size and its precision (such as 95% confidence interval) | -, missing data (95% confidence interval in placebo group) |
|  | 17b | For binary outcomes, presentation of both absolute and relative effect sizes is recommended | N/A |
| Ancillary analyses | 18 | Results of any other analyses performed, including subgroup analyses and adjusted analyses, distinguishing pre-specified from exploratory | + p. 89 |
| Harms | 19 | All important harms or unintended effects in each group | + p. 90, p.91 |
| Discussion | | | |
| Limitations | 20 | Trial limitations, addressing sources of potential bias, imprecision, and, if relevant, multiplicity of analyses | -/+ |
| Generalisability | 21 | Generalisability (external validity, applicability) of the trial findings | +p. 92 |
| Interpretation | 22 | Interpretation consistent with results, balancing benefits and harms, and considering other relevant evidence | + p.92 |
| Other information | | |  |
| Registration | 23 | Registration number and name of trial registry | - |
| Protocol | 24 | Where the full trial protocol can be accessed, if available | - |
| Funding | 25 | Sources of funding and other support (such as supply of drugs), role of funders | +p. 92 |

Table S4 Results of the quality assessment of De Deyn *et al.*^9^ by using the Preferred Reporting Items for Systematic Reviews and Meta-Analysis (PRISMA) checklist^17^

| **Section and Topic** | **Item #** | **Checklist item** | **Location where item is reported** |
| --- | --- | --- | --- |
| **TITLE** | | |  |
| Title | 1 | Identify the report as a systematic review. | -/+ p.497 Article is a pooled analysis, not stated as systematic review |
| **ABSTRACT** | | |  |
| Abstract | 2 | See the PRISMA 2020 for Abstracts checklist^17^. | -/+ p.497, does not contain all items from checklist. (e.g. eligibility criteria, risk of bias etc.) |
| **INTRODUCTION** | | |  |
| Rationale | 3 | Describe the rationale for the review in the context of existing knowledge. | + p.498 |
| Objectives | 4 | Provide an explicit statement of the objective(s) or question(s) the review addresses. | + p.498 |
| **METHODS** | | |  |
| Eligibility criteria | 5 | Specify the inclusion and exclusion criteria for the review and how studies were grouped for the syntheses. | + p.498 |
| Information sources | 6 | Specify all databases, registers, websites, organisations, reference lists and other sources searched or consulted to identify studies. Specify the date when each source was last searched or consulted. | - p. 498, states they pooled data from 3 RCTs, a 4^th^ one is not included hence its still being evaluated. However, does not state how studies were identified. |
| Search strategy | 7 | Present the full search strategies for all databases, registers and websites, including any filters and limits used. | - |
| Selection process | 8 | Specify the methods used to decide whether a study met the inclusion criteria of the review, including how many reviewers screened each record and each report retrieved, whether they worked independently, and if applicable, details of automation tools used in the process. | - |
| Data collection process | 9 | Specify the methods used to collect data from reports, including how many reviewers collected data from each report, whether they worked independently, any processes for obtaining or confirming data from study investigators, and if applicable, details of automation tools used in the process. | - |
| Data items | 10a | List and define all outcomes for which data were sought. Specify whether all results that were compatible with each outcome domain in each study were sought (e.g. for all measures, time points, analyses), and if not, the methods used to decide which results to collect. | - |
|  | 10b | List and define all other variables for which data were sought (e.g. participant and intervention characteristics, funding sources). Describe any assumptions made about any missing or unclear information. | - |
| Study risk of bias assessment | 11 | Specify the methods used to assess risk of bias in the included studies, including details of the tool(s) used, how many reviewers assessed each study and whether they worked independently, and if applicable, details of automation tools used in the process. | - |
| Effect measures | 12 | Specify for each outcome the effect measure(s) (e.g. risk ratio, mean difference) used in the synthesis or presentation of results. | + p. 500 |
| Synthesis methods | 13a | Describe the processes used to decide which studies were eligible for each synthesis (e.g. tabulating the study intervention characteristics and comparing against the planned groups for each synthesis (item #5)). | + p. 499 |
|  | 13b | Describe any methods required to prepare the data for presentation or synthesis, such as handling of missing summary statistics, or data conversions. | +p. 499, p. 500 |
|  | 13c | Describe any methods used to tabulate or visually display results of individual studies and syntheses. | + p.499 |
|  | 13d | Describe any methods used to synthesize results and provide a rationale for the choice(s). If meta-analysis was performed, describe the model(s), method(s) to identify the presence and extent of statistical heterogeneity, and software package(s) used. | + p. 499, p. 500 |
|  | 13e | Describe any methods used to explore possible causes of heterogeneity among study results (e.g. subgroup analysis, meta-regression). | + p. 499 |
|  | 13f | Describe any sensitivity analyses conducted to assess robustness of the synthesized results. | + p. 500 |
| Reporting bias assessment | 14 | Describe any methods used to assess risk of bias due to missing results in a synthesis (arising from reporting biases). | + p. 499, p. 500 |
| Certainty assessment | 15 | Describe any methods used to assess certainty (or confidence) in the body of evidence for an outcome. | + p. 500 |
| **RESULTS** | | |  |
| Study selection | 16a | Describe the results of the search and selection process, from the number of records identified in the search to the number of studies included in the review, ideally using a flow diagram. | - |
|  | 16b | Cite studies that might appear to meet the inclusion criteria, but which were excluded, and explain why they were excluded. | + p. 498 |
| Study characteristics | 17 | Cite each included study and present its characteristics. | + p. 499 |
| Risk of bias in studies | 18 | Present assessments of risk of bias for each included study. | - |
| Results of individual studies | 19 | For all outcomes, present, for each study: (a) summary statistics for each group (where appropriate) and (b) an effect estimate and its precision (e.g. confidence/credible interval), ideally using structured tables or plots. | + p. 499, p. 500, p. 501, p. 502, p. 503, p. 504, p. 505, p. 506 |
| Results of syntheses | 20a | For each synthesis, briefly summarise the characteristics and risk of bias among contributing studies. | -/+ p. 500 |
|  | 20b | Present results of all statistical syntheses conducted. If meta-analysis was done, present for each the summary estimate and its precision (e.g. confidence/credible interval) and measures of statistical heterogeneity. If comparing groups, describe the direction of the effect. | + p. 502 |
|  | 20c | Present results of all investigations of possible causes of heterogeneity among study results. | + p. 504 |
|  | 20d | Present results of all sensitivity analyses conducted to assess the robustness of the synthesized results. | + p. 504 |
| Reporting biases | 21 | Present assessments of risk of bias due to missing results (arising from reporting biases) for each synthesis assessed. | + p. 504 |
| Certainty of evidence | 22 | Present assessments of certainty (or confidence) in the body of evidence for each outcome assessed. | + |
| **DISCUSSION** | | |  |
| Discussion | 23a | Provide a general interpretation of the results in the context of other evidence. | + p. 506 |
|  | 23b | Discuss any limitations of the evidence included in the review. | - |
|  | 23c | Discuss any limitations of the review processes used. | - |
|  | 23d | Discuss implications of the results for practice, policy, and future research. | + p. 506 |
| **OTHER INFORMATION** | | |  |
| Registration and protocol | 24a | Provide registration information for the review, including register name and registration number, or state that the review was not registered. | - |
|  | 24b | Indicate where the review protocol can be accessed, or state that a protocol was not prepared. | - |
|  | 24c | Describe and explain any amendments to information provided at registration or in the protocol. | - |
| Support | 25 | Describe sources of financial or non-financial support for the review, and the role of the funders or sponsors in the review. | + p. 507 |
| Competing interests | 26 | Declare any competing interests of review authors. | - |
| Availability of data, code and other materials | 27 | Report which of the following are publicly available and where they can be found: template data collection forms; data extracted from included studies; data used for all analyses; analytic code; any other materials used in the review. | + |
